# Experimental Research of Dynamic Instabilities in the Presence of Coiled Wire Inserts on Two-Phase Flow

**DOI:** 10.1155/2013/714180

**Published:** 2013-01-10

**Authors:** Gokhan Omeroglu, Omer Comakli, Sendogan Karagoz, Bayram Sahin

**Affiliations:** ^1^Department of Mechanical Engineering, Faculty of Engineering, Bayburt University, 69000 Bayburt, Turkey; ^2^Department of Mechanical Engineering, Faculty of Engineering, Atatürk University, 25240 Erzurum, Turkey; ^3^Department of Mechanical Engineering, Faculty of Engineering and Architecture, Technical University of Erzurum, Erzurum, Turkey

## Abstract

The aim of this study is to experimentally investigate the effect of the coiled wire insertions on dynamic instabilities and to compare the results with the smooth tube for forced convection boiling. The experiments were conducted in a circular tube, and water was used as the working fluid. Two different pitch ratios (*H*/*D* = 2.77 and 5.55) of coiled wire with circular cross-sections were utilised. The constant heat flux boundary condition was applied to the outer side of the test tube, and the constant exit restriction was used at the tube outlet. The mass flow rate changed from 110 to 20 g/s in order to obtain a detailed idea about the density wave and pressure drop oscillations, and the range of the inlet temperature was 15–35°C. The changes in pressure drop, inlet temperature, amplitude, and the period with mass flow rate are presented. For each configuration, it is seen that density wave and pressure drop oscillations occur at all inlet temperatures. Analyses show that the decrease in the mass flow rate and inlet temperature causes the amplitude and the period of the density wave and the pressure drop oscillations to decrease separately.

## 1. Introduction

In many industrial systems in which boiling heat transfer exists, the flow instabilities occurring based on the boiling heat transfer cause certain parts where heat is transferred to breakdown. The fact that the system pressure, flow rate, and similar parameters create oscillations shortens the life of the process systems. It is possible to order the events decreasing the existence of the thermal systems operated by two-phase flows as thermal fatigues, mechanical vibrations, the difficulty of control caused by high transient temperatures, and the burn-out occurring on the test pipe surface [1, 2].

The main property categorising the type of two-phase flow is the shapes that interfaces occurring between two-phases take. The effect of the flow direction on these occurring shapes is highly distinctive. Two-phase flows are classified as horizontal, vertical, and inclined. The flow regimes and the characteristics occurring in horizontal, vertical, and inclined tubes are different from each other. The direction that the gravitational force affects according to the flow direction brings together the main classification [1, 3, 4]. Menteş et al. [5] and Kakaç et al. [6, 7] studied two-phase flows in different flow directions.

Considerable efforts have been made to investigate two-phase flow instabilities by researchers for many years because the instabilities and thus the oscillations shorten the life of the systems. In different types of test tubes and tubes with different cross-sections, Coleman and Garimella [8] and Leung et al. [9] investigated the instabilities of two-phase flow systems. The forced convection boiling in a horizontal tube was studied by Çomaklı et al. [10]. All types of dynamic instabilities were observed at all conducted temperatures, and the appearance boundaries of the oscillations were determined. It was observed that the instability of the system increased with the increase of the inlet temperature. Besides this trend, the periods, and the amplitudes of the pressure drop and density wave type oscillations decreased with the decreasing mass flow rate and increased with the decreasing inlet temperature. Furthermore, it was noticed that the channel length had an important effect on two-phase dynamic flow instabilities. In narrow channels, Tadrist [11] conducted an experimental study to examine two-phase flow instabilities. Parallel channel and single channel oscillations were clearly determined. Bao et al. [12] investigated experimentally the gas-liquid two-phase flow in a narrow channel. Heat transfer characteristics and pressure drop were studied for nonboiling single phase flows.

In single phase and two-phase flow systems, water is the most commonly used working fluid. Except for water, there are some other fluids and fluid mixtures. The R-11 working fluid in a two-phase horizontal boiling system was used by Çomaklı et al. [13] and Kakaç and Cao [14]. The amplitudes and the periods of the pressure drop type and the density wave type oscillations of R-11 were higher than those of water [13]. Thermal oscillations and pressure drop type oscillations occurred for all thermal power levels. The periods and the amplitudes of the oscillations increased with the increase of thermal power and inlet subcooling [14]. Oil-water mixtures were used by Poesio [15] and Sotgia et al. [16] in two-phase flow systems to analyse the combined effect of both fluids on dynamic instabilities. 

In order to investigate the two-phase flow dynamic instabilities, different approaches have been employed besides experimental methods. Homogenous and drift flow models and the Wojtan-Ursenbacher-Thome flow model were used by Moreno Quibén and Thome [17] and Kakac and Bon [18] in a horizontal test tube, and the model results were confirmed with experimental models. Yu et al. [19] investigated two-phase pressure drop, forced convection boiling heat transfer and the critical heat flux of water in a horizontal test tube. The results were successfully related to the correlations improved for different types of working fluids and boiling water in both narrow and wide channels. 

In order to enhance the heat transfer in two-phase flows, there are numerous techniques as in the single phase flows. At the head of these techniques, inserting inner elements with different geometry and configuration as turbulence promoter and surface area enhancer is the most preferred among the passive heat transfer enhancement techniques. Yılmaz et al. [20, 21] examined the heat transfer enhancement methods used in two-phase flow in detail and presented basic findings related to heat transfer enhancement in two-phase flows. The effects of enhanced surfaces on two-phase flow instabilities in a horizontal boiling system were investigated by Widmann et al. [22]. Dynamic instability types occurred in all different tube types. Çomakli et al. [4] and Karagoz et al. [23] studied the dynamic flow instabilities in a forced convection boiling system using a smooth tube and a tube with different insertions. By the addition of the insertions into the flow field, it was seen that the periods and the amplitudes of the pressure drop type and density wave type oscillations of the test tube with inner elements were higher than those of the smooth tube, and the smooth tube had a very stable structure in terms of flow instability. It was also noticed that the stability of the system increased with the decrease of the equivalent diameter for the same type enhanced surface element. In this experimental work, the coiled wire insert with two pitch ratios was used as turbulator in addition to the smooth tube in order to investigate the effects on two-phase flow instabilities. Besides the inner element and thus the pitch ratio, the effect of the inlet subcooling and the mass flow rate were also examined.

## 2. Experimental Set-Up

**Figure 1:**
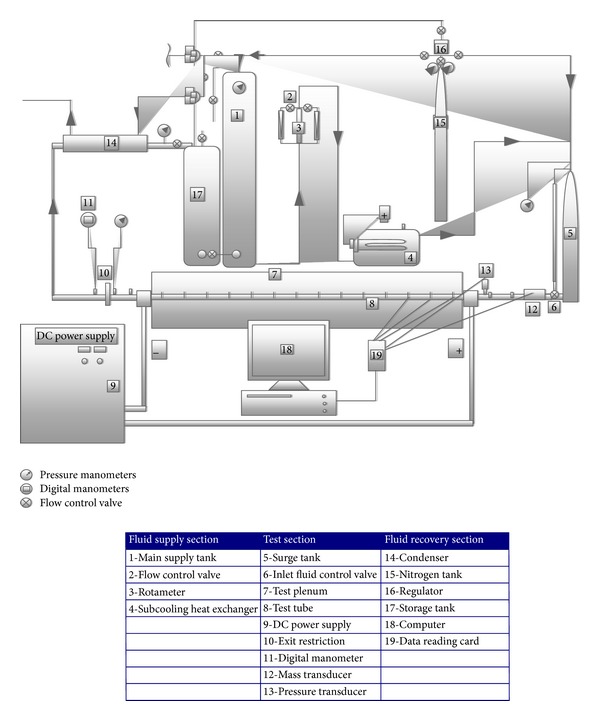
Schematic of the experimental set-up.

The experimental set-up is schematically shown in Figure 1, which has been designed to create three main dynamic instabilities (pressure drop type, density wave type and thermal oscillations). In this study, the effects of inlet subcooling, the insertion type, pitch ratio, and the mass flow rate on two-phase flow instabilities have been investigated. As seen in Figure 1, the experimental system consists of three main parts: fluid supply section, test section, and fluid recovery section. The working fluid (water) in liquid form supplied from the fluid supply section enters the circular tube and turns into a mixture of liquid and vapour by the effect of the heat input from the tube walls. The fluid that is nearly in the vapour phase at the end of the test section is sent to the fluid recovery section. This flow process goes on consistently as a close loop. 

### 2.1. Fluid Supply Section

The fluid supply section is composed of a main tank (1), flow control valve (2), flow meter (3), and heater (4). The main tank made of stainless steel stores the water used during the experiments. The cylindrical tank has 3 metres of height and 0.7 m^3^ volume rates. The flow control valve is used to set the rate of the flow to the desired level. The flow rate is controlled by two flow meters. The ranges of the flow meters is 0–400 and 0–1000 l/hour, respectively. The first flow meter is only used in small flow rates to obtain more sensitive measurements. The inlet subcooling level is remarkably important for two-phase flow experiments. Therefore, a shell-tube heat exchanger is used to send the water at the desired temperature to the test section. The working fluid is heated by electrical heaters while passing through the tube. The temperature of the fluid heated by two heaters (each heater is 4 kW) is controlled with a digital thermometer. 

### 2.2. Test Section

The test section where the dynamic flow instabilities are generated included a surge tank (5), inlet fluid control valve (6), test plenum (7), test tube (8), DC power supply (9), orifice (10), digital manometer (11), flow meter (12), and pressure transducer (13). In order to create the compressible volume for two-phase flow of water, a surge tank with 0.05 m^3^ was used. A level viewing glass, which is durable against high pressure up to 30 bar, was added to the surge tank in order to see the variations in both water and the compressible volume. The surge tank also contained a level range to measure the level and a manometer to measure the pressure inside the tank. A turbine type flow meter to measure the oscillations in water flow rate, a Bourdon type manometer to measure the pressure of the water at the tube inlet, and a pressure transducer to measure the fluid pressure oscillations at the tube inlet were installed between the surge tank and the test tube. The inlet temperature of the working fluid at the tune inlet was measured with a T-type thermocouple. An orifice plate was installed on the exit side of the tube in order to define the effects of exit restriction on flow oscillations. A Bourdon type manometer was used to measure the pressure difference caused by the orifice plate.

**Figure 2:**
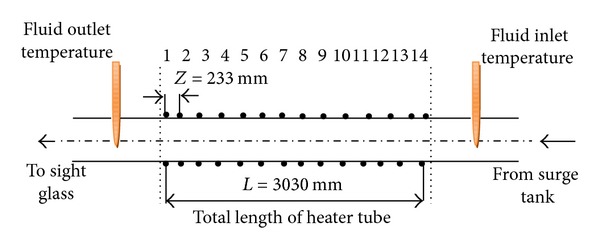
Thermocouples location.

A stainless-steel pipe of 0.017 m outer diameter, 3.4 mm wall thickness, and 3 m length was heated with uniform electrical heat input from a DC generator (24 kW power supply). To measure the oscillations of the wall temperature, 28 copper-constantan thermocouples were fixed on the outer surface of the test tube (Figure 2). Half of these thermocouples, that is, 14 thermocouples, were fixed along the top of the test tube; the other half were fixed along the bottom of the test tube. The fluid outlet bulk temperature was measured by placing another thermocouple midstream inside the tube immediately after the test section. The exit restriction (10) created the necessary pressure drop. In this system, an orifice plate was used as exit restriction and the diameter ratio of the exit restriction equal to 0.448 was used. The diameter ratio is defined as the ratio of the inner diameter of the orifice plate to the inner diameter of the tube (*β* = *d*/*di*). Downstream of the exit restriction, the exit pressure was measured with a pressure gauge. The test tube was electrically heated uniformly and insulated with glass wool that can withstand a temperature of 1000°C. The heat input was determined by measuring the current and the voltage drop across the heated section. In order to avoid floating voltage effects, the thermocouple bead was insulated from the electrically heated tube wall surface with a dab of electrically nonconductive paste. The test tube was followed by a sight glass for visual inspection of the flow. 

### 2.3. Fluid Recovery Section

After passing through the test tube, the working fluid comes into the fluid recovery section in the vapour phase. The fluid recovery section mainly consists of four components: condenser (14), nitrogen tank (15), regulator (16), and fluid storage tank (17). The working fluid, nearly all in the vapour phase at the end of the test section, is condensed with a water cooled condenser. The condensed water is sent to the storage tank. The storage water is again sent to the main tank by pressuring with the help of nitrogen gas.

### 2.4. Experimental Procedure

**Figure 3:**
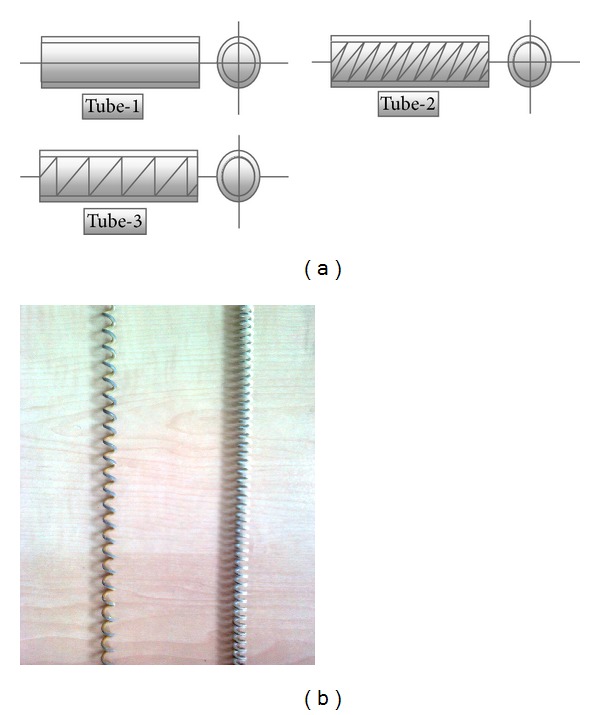
(a) Heat transfer surface augmentation elements, (b) the pictures of the elements.

The experiments were conducted in two different categories: steady and unsteady experiments. In steady experiments, the steady state characteristics were defined; likewise, the two-phase flow dynamic instabilities were investigated in unsteady experiments. In order to investigate the effects of inlet subcooling on steady and unsteady state characteristics, the experiments were conducted for three different inlet temperatures of 15°C, 25°C, and 35°C and three different test tubes under constant heat input (24 kW), system pressure (7.5 bar), and exit restriction (diameter ratio of 0.448). The first mass flow rate for each group is 110 g/s, because the flow is single phase flow at high flow rates, and the mass flow rate was decreased by 10–12 g/s slight steps to define the characteristic curve. The lowest flow rate was taken as 20 g/s in the experiments. Because of the burn-out possibility, flow rates lower than 20 g/s cannot be achieved. In Figure 3(a), the heat transfer enhancement surfaces and characteristics are presented.  Figure 3(b) shows the heat transfer enhancement elements: rings. The insertions are generally characterised with the effective diameter.  Table 1 presents the effective diameter and pitch ratios for all types of tube. The experiments were first conducted with a smooth tube (Tube-1) and then repeated for Tube-2 and Tube-3.

At the beginning of the experiments, the main tank was pressured with high pressure nitrogen gas, and the system pressure was set by the pressure regulator on the nitrogen tube. In steady state experiments, the nitrogen gas was not used, so the gas inside the surge tank was drained. As for the unsteady experiments, the surge tank was pressured with the nitrogen gas in order to create a constant compressible volume. The water level inside the tank was controlled and observed by the aid of a transparent tube level gauge. For both steady and unsteady experiments, the flow rate was set to the highest value of 110 g/s with a control valve, and to achieve the desired temperature at the tube inlet the temperature of water leaving the main tank was controlled with a digital thermostat. Later, the cooling water was sent to the condenser, and then the constant heat was applied to the tube walls by a DC power supply. Finally, the system was operated and brought to a steady state. It was decided that the steady state was achieved when no higher change than 0.5°C was observed on the test tube surface temperatures. After the steady state was reached, all measurements were taken and the same procedures are repeated for each other mass flow rates up to 25 g/s.

The fact that quick variations in the pressure value of the surge tank and water level were observed means that the oscillations began. At the unsteady experiments, the mass flow rate was decreased until the oscillation boundary was reached. In order to define the boundary where the pressure drop type oscillations break up, the periods of the oscillations were observed. The slight periods in the oscillations show the boundary where the pressure drop type oscillations finished and the independent density wave type oscillations started. By the decreasing mass flow rate, the wall temperatures were followed carefully and the creation of the thermal oscillations was provided. The experiments were suddenly halted when the burn-out began. 

### 2.5. Experimental Measurements and Uncertainties

In the experimental facility, the temperatures were measured with T-type copper-constantan thermocouples with a 0.25 mm diameter. The reading measurement of the temperature taken by the thermocouples was within ± %0.5°C. The thermocouples were placed in a copper pipe for inlet and outlet temperature readings and fixed on the tube outer wall for defining two-phase flow regimes and oscillations. An Advantech data reading card was used for conversion of the signals taken from thermocouples, pressure transducer, and flow meters, separately. The total uncertainty in readings based on chosen acquisition level was in the range of 0.1–0.5°C. 

The measured pressures in the experimental study are the pressure of the main tank, surge tank, and nitrogen tank. The pressures at the orifice inlet and outlet were also measured. The uncertainty read on the analogue manometers was ±%0.1 bar. The outlet pressure of the orifice was measured with a digital manometer. The pressure transducer was only used to measure the oscillations occurring at the test tube inlet and outlet; the pressure uncertainty of the transducer is ±%0.1 bar.

On the determination of the oscillation and the stability boundaries, the mass flow rate measurements should be taken carefully. Two flow meters having flow rate setting on itself are used to measure and set the mass flow rate. The ranges of the flow meters are 0–400 and 0–1000 l/hour, respectively. By these flow meters, the uncertainty rate is ±%0.4 l/hour. The total uncertainty of the readings taken from the turbine type flow meter used to measure the flow rate oscillations is ±%0.05 l/hour. The power rate of the DC power supply was set with the setting button on the supplier, and the heat input values were read from the digital volt and current gauges. The DC power supply was controlled within ±%0.2. 

## 3. Results and Discussion

In two-phase flow systems, the steady state characteristic curves that are used frequently are plotted by the pressure drop versus mass flow rate. These curves show the variations of pressure drop versus mass flow rate for each tube configuration. On measuring the pressure drop, the pressure drop between the surge tank and the test tube outlet is used. Also, the steady state curves depending on the inlet subcooling are plotted on defining the steady state characteristic curves. These curves are used for understanding the flow characteristics. 

The positive sloped parts of the curves that correspond to high mass flow rate values represent the single phase flow region. The first bubbles are observed at the point on which the value of the pressure drop is at a minimum with a decreasing mass flow rate and the slope of the curve is negative. On the same point, two-phase flow starts by the observation of the first bubbles. Much more vapour flows with liquid phase as long as the number of the bubbles increases in two-phase flow. This increase in the number of the bubbles causes the density to decrease in terms of the liquid phase and thus the pressure drop to increase. As a result of the fact that the mass flow rate is decreased, the parts of the curves with a negative slope up to the saturated vapour boundary have a positive slope and the pressure drop starts to decrease. On the steady state characteristic curves, the region between the dashed lines represents the unstable region where the pressure drop type (p. d. o.) and the density wave type (d. w. o.) oscillations occur at the same time; this region is called the superimposed region. The left side of the superimposed region (the left side of the dashed line on the left hand side) indicates only the density wave type (d. w. o.) flow oscillations.

**Figure 4:**
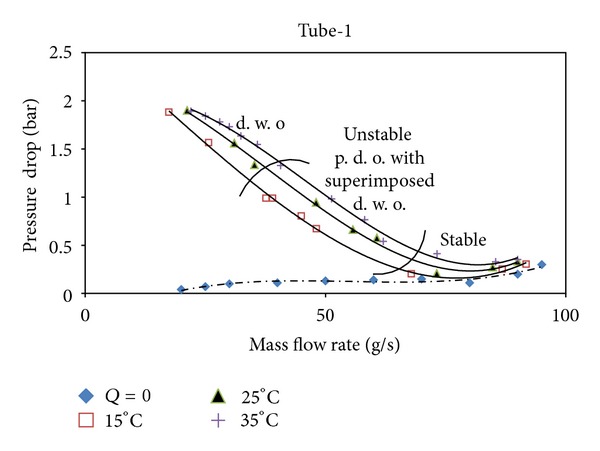
The steady state characteristic curves for Tube-1.

**Figure 5:**
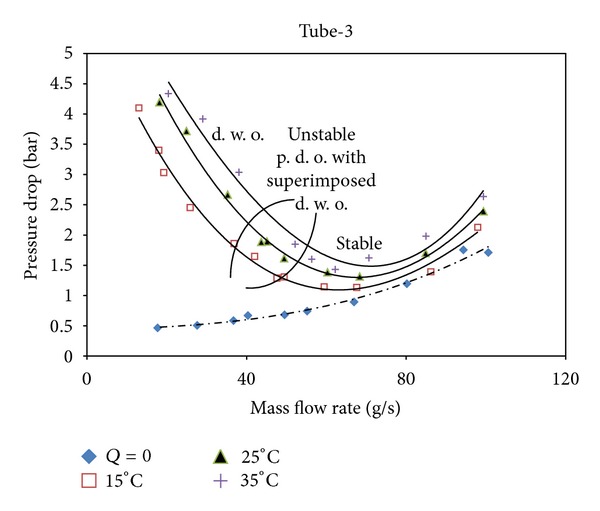
The steady state characteristic curves for Tube-3.

**Figure 6:**
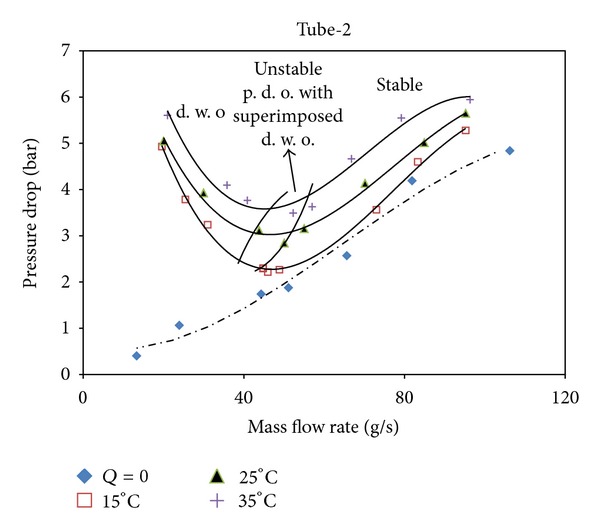
The steady state characteristic curves for Tube-2.

Using inner elements such as rings and twisted tapes in the flow field causes more friction than the smooth ones and also causes the pressure drop to increase. On the other hand, the creation of the turbulence and the removal or destruction of the boundary layer enhance the heat transfer rate. In Figures 4, 5, and 6, *Q* = 0 implies that no heat input is applied and this case presents the single phase steady state characteristics. The highest pressure drop is observed in tube-2, whereas the lowest pressure drop value is in tube-1. On the comparison of the pitch ratios, the pressure drop increases with the increase in the pitch ratio. Tubes are ordered from the highest pressure drop to the lowest value as tube-2, tube-3, and tube-1.

**Figure 7:**
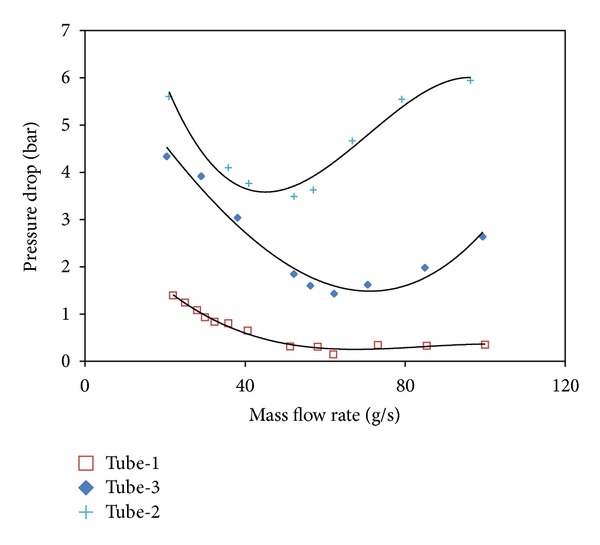
The comparison of the steady state characteristics for all tubes. *p*_in_ = 7.5 bar, *Q* = 24 kW, *T*_in_ = 25°C, *β* = 0.45.

In Figure 7, the comparison of the steady state characteristic curves for all investigated tubes are presented at *T*_*i*_ = 25°C. As shown in the graph for five different test tubes, it is observed that the pressure drop between the maximum and the minimum points of the curves increases and the points where the boiling starts slip towards lower mass flow rates. Furthermore, the increase in the inlet subcooling increases the slope of the two-phase flow region and decreases the pressure drop values with the decreasing mass flow rates. It also can be said that the system becomes more unstable as long as the negative slope angle of the steady state characteristic curves increases. As the negative slope angle of the smooth tube is lower than the tubes with inner elements, it can be concluded that the smooth tube is more stable than the other tubes. 

In two-phase flow systems, as the oscillations occurring under unsteady state conditions harm the systems, the fact that they are kept under control is highly important.  Figure 10 presents the comparison of the oscillation boundaries for all tubes. On these curves, the region on the right side of the dashed lines implies the single phase flow region on high mass flow rates, and the space between the lines implies the unsteady region where the pressure drop and density wave type oscillations occur as imposed. The left side of the middle region represents the density wave oscillations only. In the single phase flow region corresponding to the high flow rates, no oscillation is observed. By the creation of the first bubbles, the oscillations begin occurring. These oscillations are the pressure drop type (p. d. o.) and the density wave type (d. w. o.) oscillations. The beginning and the end points of the p. d. o. slip towards lower flow rates as long as the inlet subcooling rate increases. By the decrease of the inlet temperature, the single phase flow region increases and thus the system becomes more stable.

The reason why the system is more stable is the fact that the oscillations start at lower mass flow rates. The oscillations in the tubes with insertions begin at lower flow rates than the smooth tube and finish at higher flow rates. The insertions used in the horizontal tubes in which the phase separation occurs between the vapour and the liquid by the effect of the gravity disperse the vapour and cause the liquid bubbles to occur on the upper wall besides the vapour. Regarding the oscillation boundaries, the start point of the p. d. o. draws back towards the lower flow rates as long as the inlet temperature decreases. This case shows that increasing the inlet subcooling makes the system more stable for all tubes investigated. The stability boundary curves are in same manner for all tubes. The unsteady region of the flow expand, that is to say the imposed oscillation region of p. d. o. and d. w. o., grows by the increase of the gap between the boundaries. 

**Figure 8:**
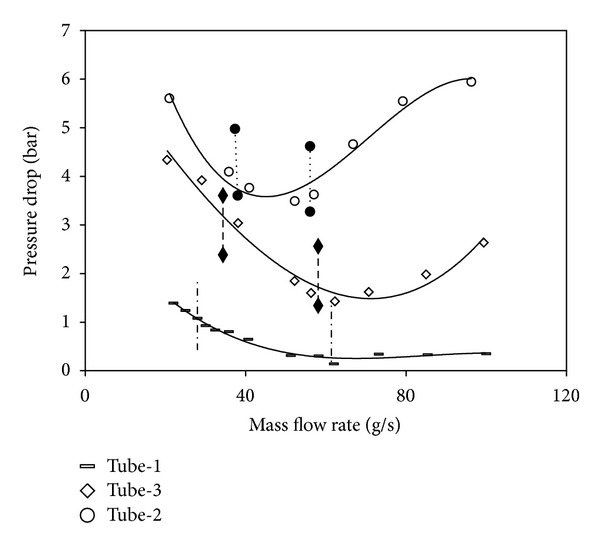
The comparison of the oscillation boundaries for all tubes *T*_in_ = 25°C.

**Figure 9:**
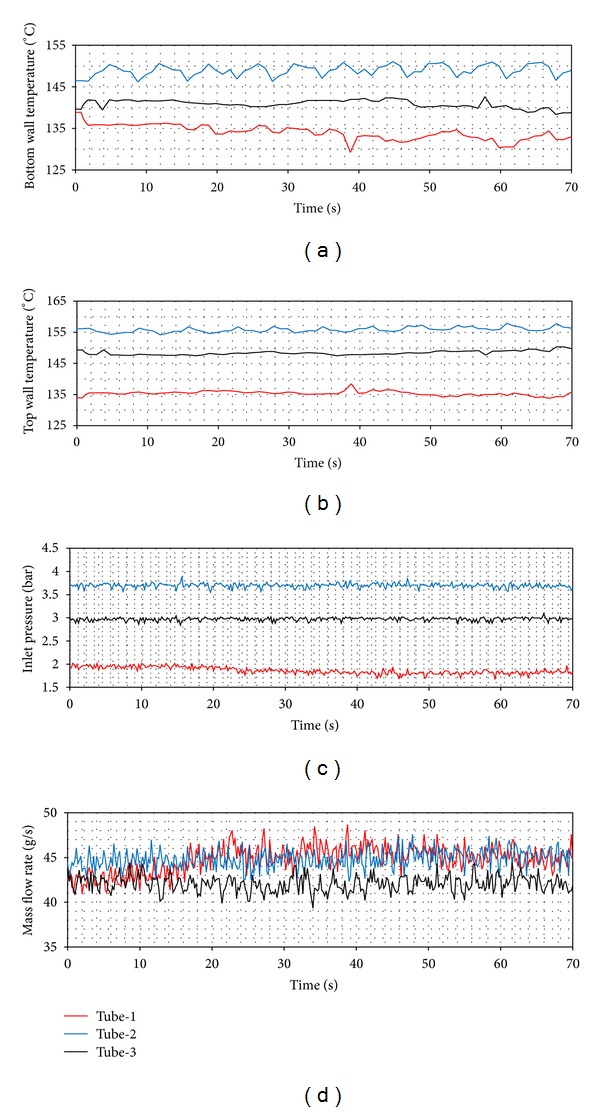
Pressure drop oscillations (*Q* = 24 kW, *T*_in_ = 15°C).

**Figure 10:**
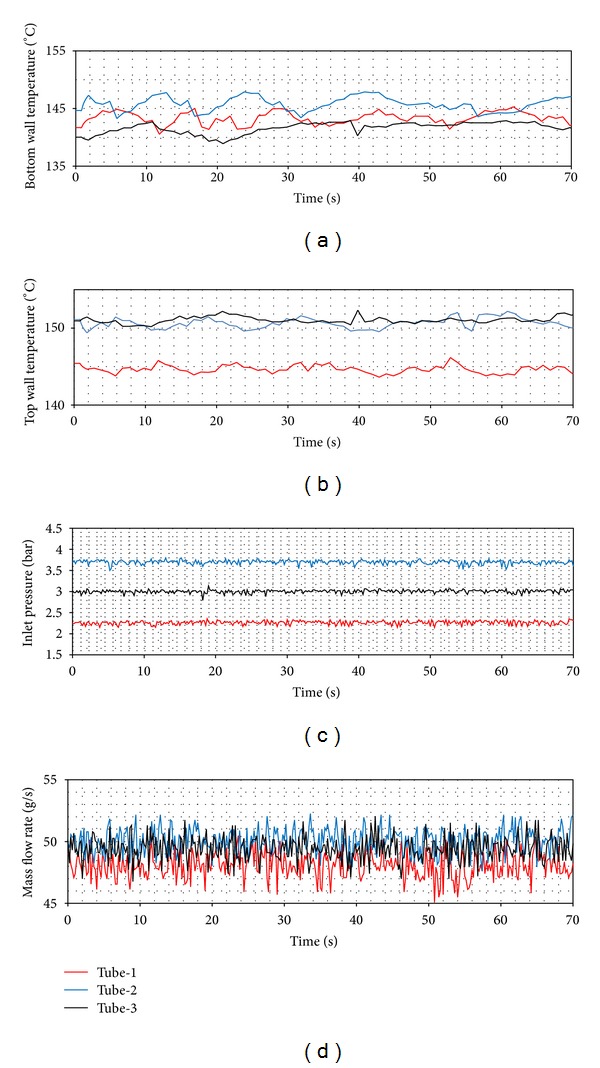
Pressure drop oscillations (*Q* = 24 kW, *T*_in_ = 25°C).

**Figure 11:**
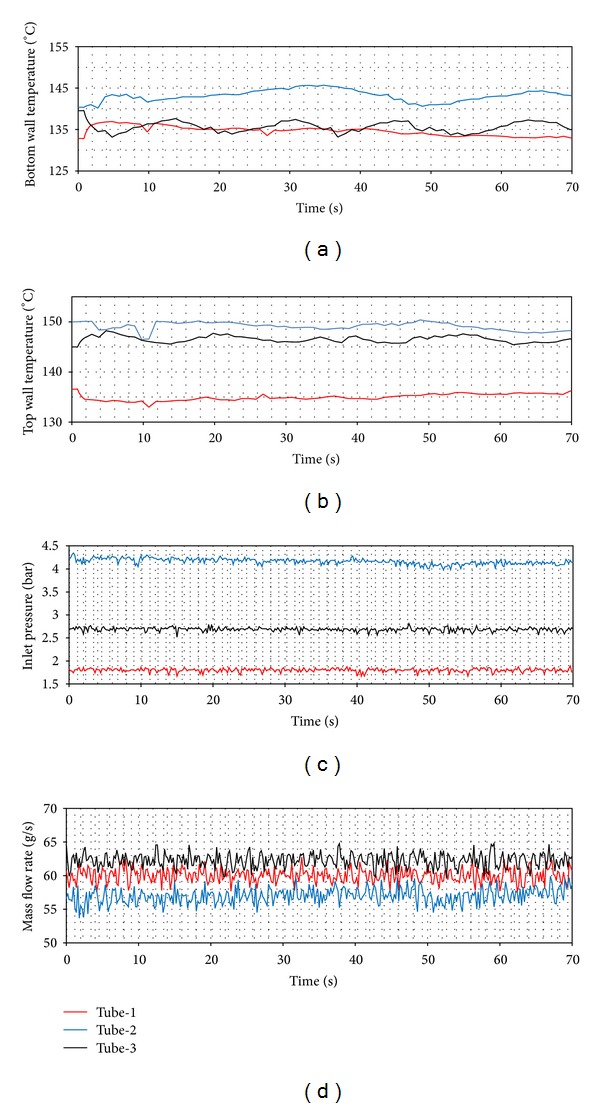
Pressure drop oscillations (*Q* = 24 kW, *T*_in_ = 35°C).

In Figure 8, it is clearly seen that tube-1 has the widest boundaries. This means that the most unstable flow occurs in tube-1. The tubes with coiled wire insertions (tube-2 and tube-3) provide a more stable flow than the smooth tube. As for the pitch ratio, the fact that the pitch increases makes the system more unstable. In Figures 9, 10, and 11, the time dependent top and bottom wall temperatures, the inlet pressure, and the mass flow rate are shown schematically. The fact that the bubbles occurring because of the too small tube diameter cover both the top and the bottom walls prevents higher temperature differences to comprise between the walls. For each ring type, the p. d. o. oscillations are observed and these oscillations cause fluctuations with big amplitudes in the inlet pressure, top and bottom wall temperatures, and the mass flow rate. Also, oscillations in the mass flow rate are observed in addition to the inlet pressure. The amplitudes of the top wall are higher than at the bottom wall. When the thermal oscillations occur, the inlet pressure and the mass flow rate oscillate at lower frequency with large amplitude.

**Figure 12:**
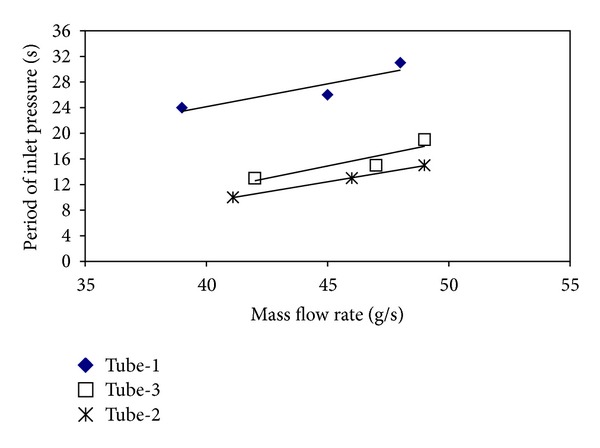
The variations in the period with the mass flow rate for all tubes (p. d. o.).

**Figure 13:**
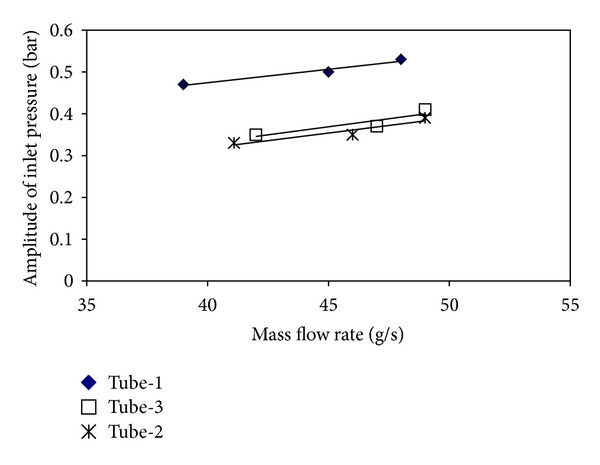
The variations in the amplitudes with the mass flow rate for all tubes (p. d. o.).

The pressure drop type flow oscillations are like dynamic instabilities occurring in unstable flows. The main effect causing pressure drop type flow oscillations to occur is the fact that a compressible volume exists. If the test section is too long, the inner compressibility is enough for the pressure drop oscillations to occur; if not, the compressible volume is provided by a surge tank located at front side of the test section. In industry, the pressure drop oscillations for the systems including two-phase flows have a large oscillation period and high oscillation amplitude. This case causes the life of the system to decrease and makes it difficult to control the flow. Therefore, the fact that the pressure drop oscillations are determined and taken under control is vitally important. In Figures 12 and 13, the effects of the mass flow rate on inlet pressure periods and amplitudes for all investigated tubes at *T*_*i*_ = 15°C are presented. For all tubes, the periods and the amplitudes belonging to the inlet pressure increase by the increase of the mass flow rate. If an order is made in terms of the periods and the amplitudes, it easily seen that the periods and the amplitudes in tube-1 (smooth tube) are higher than those of the tubes with insertions. The tubes are ordered from the highest period and amplitude to the lowest ones as tube-1, tube-3, and tube-2. According to this order, the smooth tube with the biggest effective diameter has the biggest period and amplitude, while tube-2 with the smallest effective diameter has the smallest period and amplitude. In the other words, it can be said that the period and the amplitude increase as long as the effective diameter increases. Furthermore, the periods and the amplitudes also increase with the increase in the pitch ratio. 

The density wave flow oscillations occur as a result of boiling and the hydrodynamic behaviours of the flow type. The density wave flow oscillations take place together with the pressure drop oscillations as superimposed in the region with negative slope. The pure density wave oscillations are observed as long as they move to the upper points of the negative sloped region, towards the unstable region. The period and the amplitude values of the pure density wave oscillations are lower than the pressure drop type. Furthermore, the pure d. w. o. in smooth tubes occurs at higher flow rates relative to the tubes having insertions. In two-phase flows, the separation of the phases in the smooth tubes is observed more clearly than in the tubes with insertions. 

**Figure 14:**
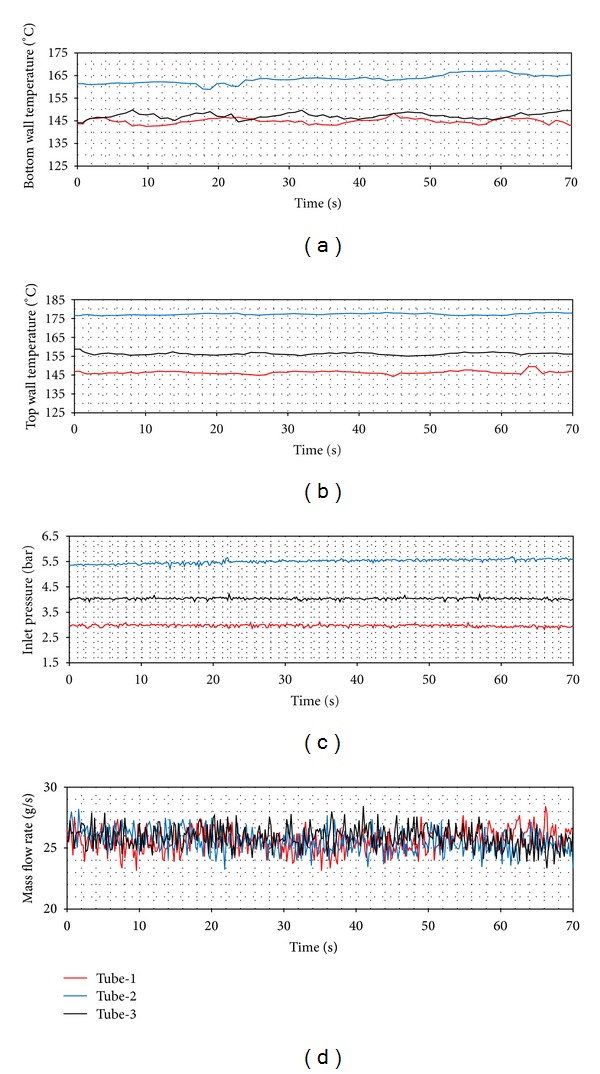
Density wave oscillations (*Q* = 24 kW, *T*_in_ = 15°C).

**Figure 15:**
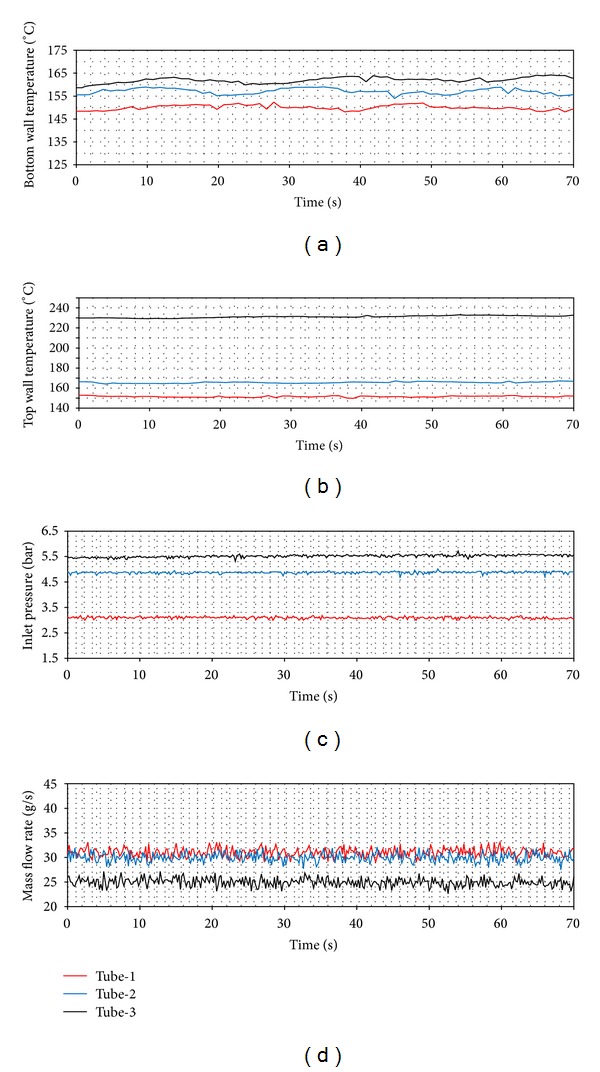
Density wave oscillations (*Q* = 24 kW, *T*_in_ = 25°C).

**Figure 16:**
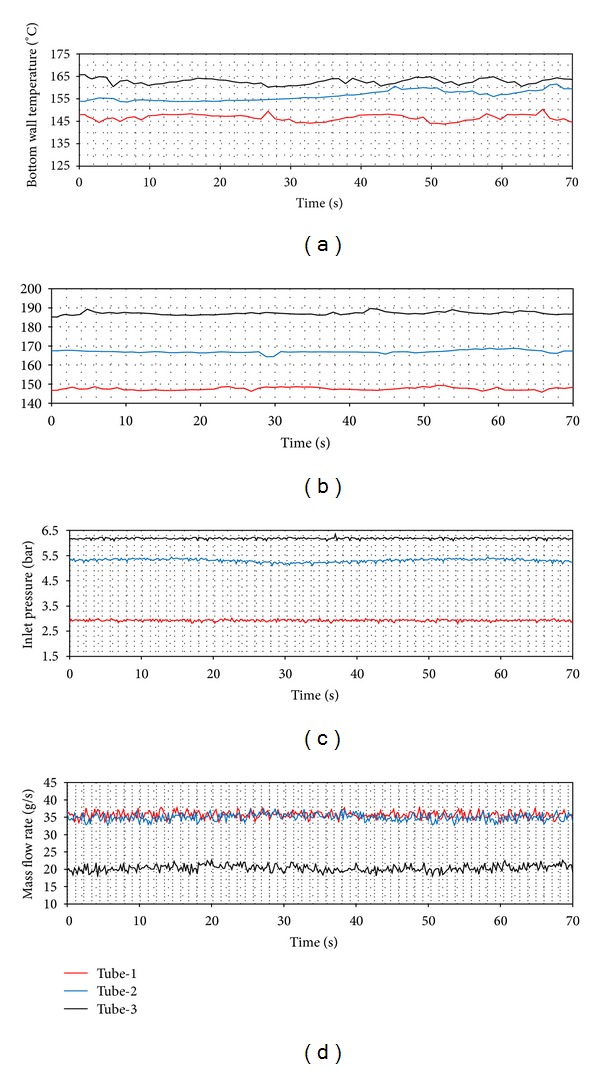
Density wave oscillations (*Q* = 24 kW, *T*_in_ = 35°C).

In Figures 14, 15 and 16, the time dependent variations of both top wall and the bottom wall, the inlet pressure and mass flow rates are presented. For each ring type, the d. w. o. oscillations are observed and these oscillations cause fluctuations with big amplitudes in the inlet pressure, top and bottom wall temperatures, and the mass flow rate. Also, oscillations in the mass flow rate are observed in addition to the inlet pressure. The amplitudes of the bottom wall are higher than at the top wall. When the thermal oscillations occur, the inlet pressure and the mass flow rate oscillate at lower frequency with large amplitude.

**Figure 17:**
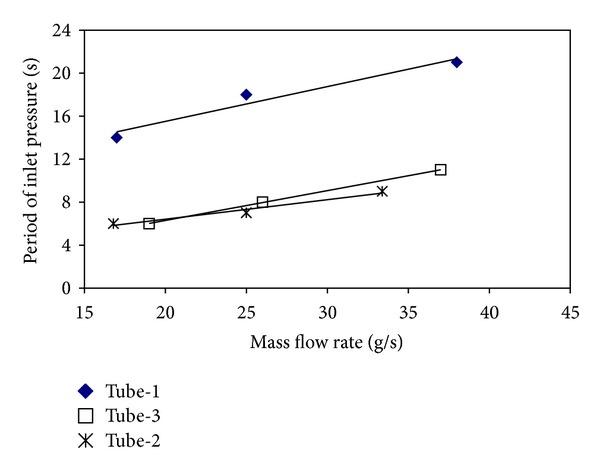
The variations of the periods with the mass flow rate for all tubes (d. w. o.).

**Figure 18:**
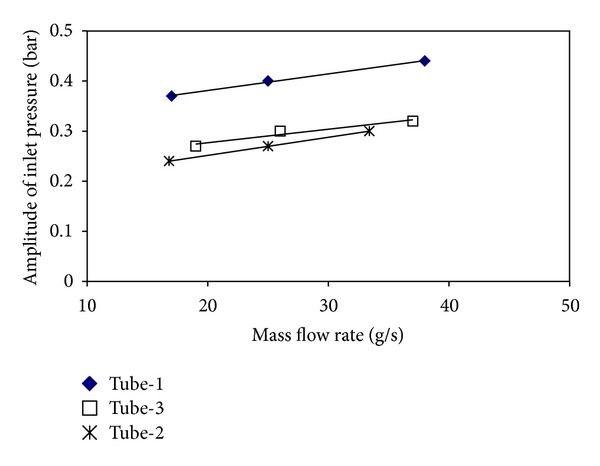
The variations in the amplitudes with the mass flow rate for all tubes (d. w. o.).

Figures 17 and 18 show the variations of the periods and amplitudes with mass flow rates. For all investigated tubes, the periods and the amplitudes increase as long as the mass flow rate increases. The amplitude and period of the smooth tube are higher than the other configurations. In terms of both the amplitude and the period, the order from the biggest to the smallest is tube-1, tube-3, and tube-2. According to the order made for pure d. w. o., the smooth tube with the biggest effective diameter has the biggest period and amplitude, and the tube-2 with the smallest effective diameter has the smallest period and amplitude. Similar to the results of pressure drop oscillations, the period and the amplitude increase as long as the effective diameter increases. Also, the periods and the amplitudes also increase with the increase in the pitch ratio. 

## 4. Conclusions

The aim of this study was to experimentally investigate the effect of the coiled wire insertions on dynamic instabilities and to compare the results with the smooth tube for forced convection boiling. The experiments were conducted in a circular tube, and water was used as the working fluid. Two different pitch ratios (*H*/*D* = 2.77 and 5.55) of coiled wire with circular a cross-section were utilised. The constant heat flux boundary condition was applied to the outer side of the test tube, and the constant exit restriction was used at the tube outlet. The remarkable conclusions are ordered as presented below.

- The rings had a higher pressure drop than the smooth tube and the pressure drop increased with the increase in the pitch ratio.
- The system became more unstable as long as the negative slope angle of the steady state characteristic curves increased. As the negative slope angle of the smooth tube was lower than the tubes with inner elements, the smooth tube was more stable than the other tubes.
- In the single phase flow region corresponding to the high flow rates, no oscillation was observed. The beginning and the end points of the p. d. o. slipped towards lower flow rates as long as the inlet subcooling rate increased. By the decrease of the inlet temperature, the single phase flow region increased and thus the system became more stable.
- The reason why the system was more stable was the fact that the oscillations started at lower mass flow rates. The oscillations in the tubes with insertions began at lower flow rates than the smooth tube and finished at higher flow rates.
- The start point of the p. d. o. drew back towards the lower flow rates as long as the inlet temperature decreased. This case showed that increasing the inlet subcooling made the system more stable for all the tubes investigated.
- The imposed oscillation region of p. d. o. and d. w. o. grew by the increase of the gap between the boundaries.
- The fact that the bubbles occurred because of the too small tube diameter covered both the top and the bottom walls prevented higher temperature differences to comprise between the walls.
- For each tube, the p. d. o. oscillations were observed and these oscillations caused the fluctuations with big amplitudes in the inlet pressure, top and bottom wall temperatures, and the mass flow rate.
- The oscillations in the mass flow rate were observed in addition to the inlet pressure. The amplitudes of the top wall were higher than those of the bottom wall.
- When the thermal oscillations occurred, the inlet pressure and the mass flow rate oscillated at a lower frequency with a large amplitude.

## Figures and Tables

**Table 1:** The tube characteristics.

Tube name	Tube characteristics	*D*_*e*_ (mm)	Pitch ratio
Tube-1	Smooth tube	8.95	—
Tube-2	Ring diameter: 1.8 mm, the pitch: 5 mm	7.96	3.33
Tube-3	Ring diameter: 1.8 mm, the pitch: 10 mm	8.44	2.77
